# Bacterial Vaginosis (BV) Candidate Bacteria: Associations with BV and Behavioural Practices in Sexually-Experienced and Inexperienced Women

**DOI:** 10.1371/journal.pone.0030633

**Published:** 2012-02-17

**Authors:** Katherine Fethers, Jimmy Twin, Christopher K. Fairley, Freya J. I. Fowkes, Suzanne M. Garland, Glenda Fehler, Anna M. Morton, Jane S. Hocking, Sepehr N. Tabrizi, Catriona S. Bradshaw

**Affiliations:** 1 School of Population Health, University of Melbourne, Melbourne, Victoria, Australia; 2 Melbourne Sexual Health Centre, The Alfred Hospital, Melbourne, Victoria, Australia; 3 Department of Molecular Microbiology, Royal Women's Hospital, Melbourne, Victoria, Australia; 4 Department of Infectious Diseases and Microbiology, Murdoch Children's Research Institute, Melbourne, Victoria, Australia; 5 Centre for Population Health, Burnet Institute, Melbourne, Victoria, Australia; 6 Department of Obstetrics and Gynaecology, University of Melbourne, Melbourne, Victoria, Australia; 7 Department of Epidemiology and Preventive Medicine, Monash University, Melbourne, Australia; Columbia University, United States of America

## Abstract

**Background:**

In recent years several new fastidious bacteria have been identified that display a high specificity for BV; however no previous studies have comprehensively assessed the behavioural risk associations of these bacterial vaginosis-candidate organisms (BV-COs).

**Methods:**

We examined the associations between 8 key previously described BV-COs and BV status established by Nugent's score (NS). We also examined the sexual practices associated with each BV-CO. We incorporated 2 study populations: 193 from a sexually-inexperienced university population and 146 from a highly sexually-active clinic population. Detailed behavioural data was collected by questionnaire and vaginal smears were scored by the Nugent method. Stored samples were tested by quantitative PCR assays for the 8 BV-COs: *Atopobium vaginae*, *Gardnerella vaginalis*, *Leptotrichia* spp., *Megasphaera* type I, *Sneathia* spp., and the Clostridia-like bacteria BVAB1, BVAB2 and BVAB3. Associations between BV-COs and BV and behaviours were examined by univariate and multivariable analyses.

**Results:**

On univariate analysis, all BV-COs were more common in BV compared to normal flora. However, only *Megasphaera* type I, BVAB2, *A. vaginae* and *G. vaginalis* were significantly independently associated with BV by multivariable analysis. Six of the eight BV-COs (*Megasphaera* type I, BVAB2, BVAB3, *Sneathia*, *Leptotrichia* and *G. vaginalis*) were rare or absent in sexually-unexposed women, and demonstrated increasing odds of detection with increasing levels of sexual activity and/or numbers of lifetime sexual partners. Only *G. vaginalis* and *A. vaginae* were commonly detected in sexually-unexposed women. *Megasphaera* type I was independently associated with women-who-have-sex-with women (WSW) and lifetime sexual partner numbers, while unprotected penile-vaginal-sex was associated with BVAB2 detection by multivariate analysis.

**Conclusions:**

Four of eight key BV-COs were significantly associated with BV after adjusting for the presence of other BV-COs. The majority of BV-COs were absent or rare in sexually-unexposed women, and associated with increasing sexual exposure, suggesting potential sexual transmission of BV-COs.

## Introduction

Bacterial vaginosis (BV) is an enigmatic condition and despite over 50 years of medical research, its aetiology and pathogenesis remains unknown. It is the commonest cause of vaginal discharge globally [1], [2], is associated with adverse pregnancy outcomes [3], [4], [5], [6] and enhancement of heterosexual HIV transmission [7]. There is debate as to whether BV is caused by a single bacterial agent or whether it has a polymicrobial aetiology. *Gardnerella vaginalis* has been the bacterium most intensively studied in relation to BV; however it is not specific for BV and is commonly present in women with normal vaginal flora although usually in lower numbers than in those with BV [8]. Recent studies have shown that a biofilm, predominantly comprised of *G. vaginalis* and *Atopobium vaginae* is associated with BV [9], and that the presence of these two organisms together in high copy numbers have high sensitivity (95%) and specificity (99%) in predicting BV [10]. However, the advent of molecular screening has also identified a number of fastidious, primarily anaerobic bacteria, in association with BV [11], [12], [13], [14], [15], [16], [17], including the Clostridia-like bacteria vaginosis-associated bacteria (BVAB) 1, 2 and 3, the closely related lactic-acid producing *Leptotrichia* and *Sneathia* genera, and the uncultivated *Megasphaera*-like phylotype (termed type I) [13]. In particular, Fredricks found that while no one bacterial vaginosis-candidate organism (BV-CO) was universally present in BV, the presence of either BVAB2 or *Megasphaera* type I had a sensitivity of 100% and specificity of 91.3% in predicting BV. The causal role of these BV-COs remains to be established and there are no published reports on their behavioural associations and whether they are sexually transmitted.

Observational evidence shows a strong association between sexual activity and BV [18], [19], [20], [21], [22]. In a recent meta-analysis BV was associated with recognized STI risk factors such as multiple sexual partners and condoms were protective against BV [21]. We have recently reported that BV is absent in women with no history of sexual activity with another individual, importantly this study considered both coital and non-coital sexual activity in their classification of sexual exposure [22], [23]. There are data to also suggest transmission of BV may occur between women [19], [21], [24], [25], [26], [27]. Women-who-have-sex-with-women (WSW) have consistently demonstrated higher BV prevalence rates than exclusive heterosexually active women, and consistent with the concept of WSW transmission, studies of female-female monogamous couples demonstrate high concordance for BV (73–95%) [24], [28]. However, there is some evidence against the sexual transmission of BV. Studies testing the impact of male partner treatment have failed to consistently reduce BV recurrence in women [29], [30], [31], [32], and some studies have identified BV in women who have not engaged in penile-vaginal sex [22], [33], [34].

Using samples from two published studies that collected detailed behavioural data and included women with a broad range of sexual experience including sexually-naive women, we sought to examine the relative strength of associations between the aforementioned key BV-COs and BV status established by Nugent's score (NS), and to establish the behavioural characteristics associated with each BV-CO.

## Methods

### Ethics statement

The Human Research and Ethics Committee of the University of Melbourne approved both studies incorporated into this research. Informed written consent was obtained from all participants involved in the study.

### Study populations and recruitment methods

In order to obtain a study population with a broad range of sexual exposure from no history of sexual activity to high levels of sexual activity, we combined populations from two published studies: the FUSS (Female University Student Study) [22], and a clinic population of 342 women attending Melbourne Sexual Health Centre (MSHC) with symptoms of vaginal discharge or odour from July 2003–August 2004 [19]. For the FUSS study, women aged between 17–21 years attending the University of Melbourne, from March–July 2008 were eligible to enrol. Advertising material was placed in the university orientation handbook and on posters throughout the university. Study staff also handed out advertising material at the university campus. Advertising material directed interested women to the study website (www.mshc.org.au/fuss/), which described the study in detail, and provided investigators' contact details. Research investigators provided a detailed explanation of the study upon contact, and a record was kept of all individuals who contacted investigators.

FUSS contained 3 groups of women: sexually-inactive women with no history of any sexual-contact with another person, women with a history of non-coital contact but no penile-vaginal sex, and women with a history of penile-vaginal sex. Overall the FUSS population had limited sexual exposure. The clinic population was recruited at MSHC by clinicians and the participants were sexually-experienced women with higher numbers of lifetime partners. This group was included to act as a comparison group to the low risk FUSS population, to extend the breadth of sexual exposure in the overall study population and to provide generalisable findings to past studies. Most studies of the association of BV-COs with BV have been in women recruited from higher prevalence populations [15], [17], [35], [36].

### Clinical methods

Participants in both studies completed questionnaires recording demographic, medical, sexual partnership and behavioural data. In FUSS, these data were collected via self-collected paper-based questionnaires with the option of online questionnaires, whereas in the clinic study women self-completed hard copy questionnaires. Questions were asked in a standardized way across both studies to allow these data to be aggregated where necessary. Both studies used the Nugent method to diagnose BV on Gram-stained vaginal smears. In FUSS they were self-collected, while they were clinician-collected in the clinic study. FUSS participants received clear diagrammatic instructions regarding self-collecting vaginal swabs and smears. Self-collected smears have been shown to be equivalent to practitioner-collected samples in published studies [37], [38] and have been extensively used in BV research.

All vaginal smears were scored using the Nugent method by an experienced microscopist with no access to the behavioural data; a Nugent score (NS) of 0–3 was graded as normal flora (NF), 4–6 as intermediate flora, and 7–10 as BV. Ten percent of slides were re-read by a second microscopist, who was blind to the original results. If consensus was not reached, a third microscopist assessed the slide and a discussion followed until agreement was reached.

Overall, 193 samples from FUSS were analysed including all samples with a NS of 7–10 (n = 24), all samples from women who reported no history of penile-vaginal sex ever (n = 82), and an equivalent random sample of women who reported penile-vaginal sex ever (n = 87). From the clinic study, 146 random samples were selected from participants reporting >10 lifetime partners (male and female partners combined). The two study populations were combined for this analysis in order to provide a uniquely diverse population of women that ranged from sexually-inexperienced (FUSS) to highly sexual experienced (clinic study). Having behavioural data and clinical samples from women with a broad range of sexual exposure was considered integral to investigating the associations between BV-COs and sexual activity. Only women with normal flora and BV were included in this analysis and women with intermediate flora were excluded. Women who were pregnant, HIV infected, postmenopausal, or non- fluent in English were excluded from participating in either study. All swabs samples used in this study were stored at −80°C until processing. The Human Research and Ethics Committee of the University of Melbourne approved both studies.

### Molecular analysis methodology

Eight BV-COs were selected including *Atopobium vaginae*, *Gardnerella vaginalis*, *Leptotrichia* spp., *Megasphaera* type I, *Sneathia* spp. and the Clostridia-like bacteria BVAB1, BVAB2, and BVAB3; cost limited the inclusion of other BV-COs. *Lactobacillus crispatus* was also included as a key indicator of normal vaginal flora status. Total DNA was extracted from 200 µl volumes of the resuspended vaginal swab samples using the MagNA Pure LC system (Roche Diagnostics) with the DNA Isolation Kit I protocol, eluting in a final volume of 100 µl with DNA stored at −20°C until testing. Included in each DNA extraction run were controls that included human A549 cells and DNA-free phosphate buffered saline. To assess inhibition, sample adequacy and integrity, real-time PCR amplification and detection of the human β-globin gene was carried out as previously described [39]. Sample DNA was then subjected to a series of quantitative PCR (qPCR) TaqMan-based assays, targeting various bacterial 16S rRNA gene targets. All assays were performed on the LC480 real-time instrument (Roche Diagnostics) using 5 µl DNA in a 20 µl reaction. Assays developed by Fredricks et al. [40] were used to detect *A. vaginae*, *L. crispatus*, *Leptotrichia* spp., *Megasphaera* type I, and the Clostridia-like bacteria BVAB1, BVAB2, and BVAB3 with the detection for *Leptotrichia* spp. and *Sneathia* spp. carried out as separate reactions. *G. vaginalis* detection was determined by an assay by Zariffard et al. [41]. Positive controls consisting of extracted DNA from culture or plasmids were included in each run as well as appropriate no template controls.

### Statistical analysis

The associations between BV-COs and BV were assessed by univariate and multivariate analyses using logistic regression. The presence of BV-COs according to sexual exposure variables were examined using Chi square tests or Fisher's exact where appropriate. To investigate the association between behavioural risk factors and BV-COs, a multivariate logistic regression model was constructed, including variables with a significance of p<0.05 from the univariate analysis and key risk factors for BV from published literature. These included: number of lifetime vaginal sexual partners (LSP), smoking, age, frequency of vaginal sex and frequency of receptive oral sex and unprotected vaginal sex (UPVSI) in the last 12 months. Samples with intermediate flora (Nugent's score 4–6) were not included in this analysis and all analysis was undertaken using PASW software (version 18).

## Results

Overall, 339 specimens were analysed; 193 from FUSS and 146 from the clinic cohort. Among the study population, 233 women had NF and 106 women had BV. Table S1 describes the demographic and behavioural characteristics of the study populations. As expected, more clinic-based women were older, more sexually-experienced, more likely to smoke cigarettes (p<0.001), but they were less likely report sex toy use (p = 0.02) than FUSS participants. The study populations were similar with respect to other reported practices including oral contraception use and douching.

### Associations between candidate organisms with bacterial vaginosis and normal flora

The association between detection of BV-COs in BV and NF were examined, Table S2. All BV-COs except BVAB1 were significantly more common in women with BV than NF; while BVAB1 was absent in NF and uncommon in BV. *L. crispatus* was significantly more common in NF than BV. In a logistic regression model, *Megasphaera* type I demonstrated the strongest independent association with BV of the 8 BV-COs [Adjusted Odds ratio (AOR) = 15.4; 95% CI 5.9–40.2, p<0.001, Table S2]. Three other BV-COs: BVAB2, *A. vaginae* and *G. vaginalis* were also significantly independently associated with BV (range of AORs = 4.1 to 15.3, Table S2), and although the AORs for BVAB3, *Leptotrichia* and *Sneathia* exceeded 1.0, the association was not statistically significant once adjusted for the other BV-COs. Despite having a protective effect against BV, the additional of *L. crispatus* to the adjusted analysis did not significantly change the associations shown in Table S2 (data not shown).

### Associations between sexual exposure and BV-COs in women with normal flora and BV

The association between BV-COs and differing degrees of sexual exposure was examined separately in women with NF and BV using two measures: reported sexual exposure categories and numbers of lifetime sexual partners (LSP), Table S3. Sexual exposure was classified according to three categories: i) no sexual contact with another individual, ii) non-coital sex only and/or 100% condom use for all vaginal sex ever and iii) unprotected vaginal sex ever. Non-coital sex and 100% condom use were combined to restrict the number of total categories and we found the women who reported 100% protected sex were behaviourally closer to those with only non-coital exposure with regard to sexual risk behaviours. Number of LSP was classified as 0, ≤10 and >10. Importantly, six of the eight BV-COs: *Sneathia*, BVAB1, BVAB2, BVAB3, *Leptotrichia* and *Megasphaera* type I, were either absent or rare in women with no reported history of sexual exposure, whereas *G. vaginalis* was detected in 28% and *A. vaginae* in 71% of sexually-unexposed women. Most of the BV-COs demonstrated an increase in detection with increasing sexual exposure measured by either the 3 exposure categories or by increasing number of lifetime sexual partners in NF and/or BV (Table S3). *A. vaginae* demonstrated least association with sexual risk with no association found with increasing sexual exposure categories in normal flora, a reverse association with lifetime partners in normal flora, and only a trend towards an association with increasing sexual exposure and BV.

### Associations between BV sexual behavioral risk factors and BV-COs

To further investigate the epidemiology of BV-COs, the association of BV sexual risk factors with each BV-CO was examined. BV risk factors included in the analysis included LSP number, UPVSI in the last 12 months, reporting a female partner (WSW) in the last year, age, smoking and frequent penile-vaginal sex and frequent receptive oral sex (>once weekly). The associations between each of these risk variables and each BV-CO was examined separately and adjusted for BV status (Table S4).

Univariate analysis demonstrated that *Sneathia*, *Leptotrichia*, *Megasphaera* type I, *G. vaginalis*, BVAB2 and BVAB3 were significantly associated with having >10 LSP or UPVSI or both factors after adjustment for BV status. Frequent sexual activity (vaginal and/or receptive oral) was related to increased detection of *Sneathia*, *Leptotrichia*, *G. vaginalis*, and BVAB3, while *Megasphaera* type I and *Leptotrichia* were significantly associated with WSW status. BVAB1 was uncommon and no associations could be examined with meaningful statistical power (Table S4). The association between BV-COs and other behavioural and demographic variables such as anal sex, douching, oral contraceptive pill, country of birth, circumcision status of partner and past history of BV were also examined but there were no significant associations.

### Behavioural associations with Megasphaera type I, G. vaginalis, BVAB2 and A. vaginae by multivariate analysis

As *Megasphaera* type I, *G. vaginalis*, BVAB2 and *A. vaginae* were significantly associated with BV status after adjustment for the presence of other BV-COs, we further explored the behavioural associations with these four BV-COs by multivariate analysis. Variables entered into the model included LSP number, UPVSI in the last 12 months, reporting a female partner (WSW) in the last year, age, smoking, frequent penile-vaginal sex and frequent receptive oral sex (>once weekly) and BV status, Table S5. By multivariable analysis *Megasphaera* type I was independently associated with WSW (AOR = 4.4; 95%CI 1.2–16.4, p = 0.03) and having more than 10 LSP (AOR = 2.9; 95% CI 1.0–8.3, p = 0.04), and BVAB2 showed a strong independent association with unprotected penile-vaginal sex in the last 12 months (AOR 22.5; 95% CI 2.6–196.0, p = 0.005); while no specific behaviours remained significantly associated with *G. vaginalis* or *A. vaginae* by multivariate analysis.

## Discussion

Our paper explores the association between eight key BV-COs with BV and examines their sexual risk factors; Table S6 summarizes our major findings. Seven of the eight BV-COs demonstrate a strong unadjusted association with BV, and confirm other studies' findings [8]–[14]. Four of the eight BV-COs (*Megasphaera* type I, BVAB2, *A. vaginae* and *G. vaginalis*) were significantly associated with BV after logistic regression analysis, while BVAB3, *Sneathia* and *Leptotrichia* spp. were not, and BVAB1 was too uncommon to meaningfully comment on. Six of the eight BV-COs demonstrated significant associations with increasing sexual exposure (*Megasphaera* type I, BVAB2, BVAB3, *Sneathia* spp., *Leptotrichia* spp. and *G. vaginalis*), and only two BV-COs, *A. vaginae* and *G. vaginalis*, were commonly detected in women with no sexual experience. Our findings suggest that there may be a gradient of importance among the eight BV-COs based on their strength of association with BV and their epidemiological association with increasing sexual exposure. The finding that the majority of BV-COs were absent or rare in sexually-unexposed women and associated with increasing sexual exposure suggest sexual transmission of BV-COs is occurring.

For agents to be causative of BV one would anticipate that they would be absent in NF and universally present in BV; no single BV-CO in our study satisfied both these criteria. We have shown, however, that *Megasphaera* type I and BVAB2 were both strongly associated with BV and were also rare or absent in sexually-naive women. The suggestion that *Megasphaera* type I and BVAB2 are key BV-COs is supported by Fredricks et al. who showed that the combination of BVAB2 and/or *Megasphaera* type I conferred the best BV diagnostic predictability for BV (sensitivity 98.8%, specificity 88.5%) [15].

The other BV-COs which were significantly associated with BV were *G. vaginalis* and *A. vaginae*, bacteria known to be integrally related to BV disease; however these BV-COs were also detected at lower loads in women with NF (*data not shown*). *G. vaginalis* has long been implicated in the development of BV and recent published findings have suggested that *G. vaginalis* biofilms may be critical in BV pathogenesis and symptomatology [42], [43]. Our results demonstrating that *G. vaginalis* was ubiquitous in women with BV, and further analysis indicating a 6–7 log_10_ greater bacterial load in BV than NF (*data not shown*), are consistent with historical findings [10], [44]. Our data also show that *G. vaginalis* was relatively common in truly virginal women (prevalence 28%); however the prevalence of *G. vaginalis* increased dramatically with numbers of sexual partners, indicating that sexual activity or transmission contributes to increasing detection of *G. vaginalis*. Studies using enzyme assays to identify *G. vaginalis* biotypes and genomic comparisons of *G. vaginalis* isolates suggest particular strains of *G. vaginalis* may have differing pathogenic properties, such as biofilm formation [45]. Whether differing biotypes of *G. vaginalis* are present in sexually-inexperienced compared to experienced women is not known and would be of considerable interest.

*A. vaginae* was independently associated with BV and whilst also commonly seen in those with NF (62%), it was generally at low loads (<4 log_10_, *data not shown*). The relatively high prevalence of *A. vaginae* in NF samples was similar to that of Menard et al [10] at 69%, but significantly higher than a previous publication that detected *A. vaginae* in 12% of NF [46]. A key difference is that in the current study, our assay used primers described by Fredricks et al. [40] which target a 81-bp region of the 16S rRNA gene, whilst the assay in the earlier publication targeted a 430-bp region [47]. Generally, the longer amplicon assay displays relatively reduced sensitivity, and the difference in sensitivities between these two assays is discussed in detail by Menard [10]. *A. vaginae*, like *G. vaginalis*, was detected in sexually-inactive women, but unlike *G. vaginalis*, *A. vaginae* showed no significant association with increasing sexual exposure.

Importantly, our data seem to indicate that *Leptotrichia* and *Sneathia* spp. are not associated with BV once adjusted for the presence of other BV-COs, and their strong relationship with increasing sexual risk was seen similarly in women with NF and BV. This could be important, because if a BV-CO is sexually transmitted but not associated with disease causation, then one would expect to see increasing prevalence with increasing sexual risk equally in both BV and NF. This was the case for *Sneathia* and *Leptotrichia* spp. but not for any of the other BV-COs. Collectively these findings suggest that *Sneathia* and *Leptotrichia* spp. could just be epidemiologically associated with BV, or sexually transmitted “passengers” rather than being involved in the development of BV; a similar scenario to that of some *Ureaplasma* and *Mycoplasma* spp. in NGU [48]. However, as *Sneathia* and *Leptotrichia* spp. were detected in 74 and 73 percent of BV cases in our study respectively, their role in symptom/disease pathogenesis, especially in a polymicrobial disease context, cannot be excluded. Future work involving the exploration of metabolic interactions between BV-COs will assist in further understanding the contribution of these organisms to the development of BV.

Our data show that *Megasphaera* type I may have distinctive behavioural risk factors. Although we only had 12% of participants with a female partner in the last 12 months, these women demonstrated a strong association with *Megasphaera* type I detection. It is important to note that WSW have greater BV population prevalence than other women [21], [24], [27], [49], [50] and the high prevalence of BV in WSW has to date remained unexplained. We note with interest that Fredricks et al. [15] found *Megasphaera* type I detection was highly sensitive for BV (94.5; 95%CI 86.7–97.9) in a population of women with a history of sexual contact with another woman. In our study *Megasphaera* type I was 91.3% (95%CI 70.4–98.4) sensitive for BV in WSW, but only 72.2% (95%CI 61.2–81.2) sensitive for BV in non-WSW. It is possible that *Megasphaera* type I may be an important BV-CO in the development of BV in WSW, and that WSW with BV differ in their microbiological BV profile to other women. Clearly larger prospective studies are needed to better understand this relationship, but an association between *Megasphaera* type I and WSW to our knowledge has not been previously described.

This study has several limitations. We used a study population that combined samples from two previous studies collected at different time points. One group was a symptomatic clinic-based population; the other a lower risk university population. By combining these populations investigators were able to obtain a uniquely diverse population of women who ranged from sexually-inexperienced to highly sexually-experienced, and we considered this necessary to be able to comprehensively address the contribution of BV-COs to sexual activity. Prior published studies investigating the association of BV-COs with BV have not contained women with limited or no sexual exposure. The fact that these populations substantially differ in their risk profile, however, is also limitation of the study. We have adjusted for lifetime sexual partner number in the multivariable analyses to control for the key differences in sexual exposure in the study groups, and although we describe known differences in the two study populations not all differences between the groups may not be accounted for. Questions were asked in a standardized way in both studies to allow these data to be aggregated where necessary, and self-collected paper-based questionnaires were used for both studies with the option of an identical self-completed online questionnaire for FUSS (40% of FUSS participants used online surveys). Self-sampling was employed in FUSS and clinician-collected samples were used in the clinic study; published studies confirm equivalency of self-collected to clinician-collected samples for the diagnosis of BV, and excellent agreement has been reported between self-collected versus clinician-collected swabs for molecular quantification (qPCR) of BV-associated bacteria [37], [38]. Specimens were stored in the same way in both studies as whole samples at −80°C, and we confirmed the integrity of stored samples in both studies.

Due to funding limitations, our use of molecular methods to characterise the vaginal bacterial communities was limited to a representative panel of eight of the most promising and specific BV-COs, acknowledging there is an increasing number of BV-COs being cited in the literature, particular from next-generation sequencing projects [51], [52]. Further research should include a greater number of BV-COs and in particular more *Megasphaera* spp. including the type II phylotype. Our data was cross-sectional, therefore we have no capacity to comment on temporal relationships between the detection of BV-COs and sexual behaviour, and we can only make inferences regarding BV causality and pathogenesis. Due to the complexity of the current analyses in this manuscript, we also did not include possible metabolic interactions between species in this study, and have limited ourselves to exploring the associations between individual BV-COs and behaviour and BV disease status. Given the polymicrobial nature of BV, assessing for metabolic interactions is the subject of further research by investigators. Another limitation of cross-sectional data is that fluctuations in vaginal flora are known to occur rapidly and to be influenced by various factors including hormonal variation and day-to-day behaviours [53]., clearly longitudinal studies with frequent sample collection are required to further understand the complex relationship between BV-COs and development of BV. As with all BV research, the lack of a gold standard test hampers our ability to explore specific disease associations. We excluded any women with intermediate vaginal flora (NS4-6) in an attempt to minimize the possible misclassification of flora caused by a cross-sectional ‘snapshot’ design in an actively changing environment. It is important to also note that this data cannot be used to determine the population prevalence of any of the BV-COs as the sampling method was not appropriate for this.

The strengths of our study design include the systematic objective assessment using Nugent's method of all vaginal smears by an experienced microbiologist and 10% being re-scored by an alternative microscopist. Not all studies examining the role of BV-COs in BV have applied methods such as the Nugent method to classify flora objectively and some have used more subjective clinical descriptions and/or patient history [12], [14], [16]. Another important strength of our research is that highly detailed behavioural data was collected and the FUSS study was quite unique, including women with a broad-range of sexual experience, in particular a considerable number with no reported sexual exposure to another person or limited non-coital exposure only. Previous BV study populations that have examined BV-COs have recruited women from STD clinics or women from high prevalence populations. The inclusion of sexually-naive women in our study, in addition to comprehensive, confidential sexual behaviour data from participants, has enabled us to explore specific sexual practices and their associations to individual BV-COs in considerable depth.

### Conclusion

These study data contribute to the developing collective understanding of BV-related bacteria and their role in BV pathogenesis. *Megasphaera* type I, BVAB2, *A. vaginae* and *G. vaginalis* were significantly associated with BV after adjusting for detection of other BV-COs; however, BVAB3, *Sneathia* and *Leptotrichia* spp. showed no independent association with BV, and BVAB1 was absent in NF and uncommon in BV. Our data show that six of the eight BV-CO were rare or absent in sexually-inactive women: *Sneathia* spp., *Leptotrichia* spp., *Megasphaera* type I, BVAB1, BVAB2 and BVAB3. *G. vaginalis* and *A. vaginae* were the only BV-COs detected commonly in sexually-inexperienced women. After multivariate-analysis *Megasphaera* type I was independently associated with WSW and lifetime sexual partner numbers, while unprotected vaginal sex was strongly related to detection of BVAB2. The finding that the majority of BV-COs were absent or rare in sexually-unexposed women, and associated with increasing sexual activity with other individuals, suggests sexual transmission of BV-COs is occurring. Longitudinal studies of BV and BV-COs, and consideration of metabolic interactions between BV-COs, will further elucidate the complex microbiology involved in the development of BV in different populations.

## Supporting Information

Table S1 **Demographic and behavioural characteristics of study population.**
(DOC)

Table S2 **Bacterial vaginosis candidate organisms in women with normal flora and BV.**
(DOC)

Table S3 **Associations between bacterial vaginosis candidate organisms and level of sexual exposure in women with normal flora and BV.**
(DOC)

Table S4 **Odds ratios for BV-COs behavioural risks factors adjusted for BV status (n = 339).**
(DOC)

Table S5 **Logistic regression analysis for BV and BV risk factors and** ***Megasphaera*** **type I,** ***G. vaginalis*** **, BVAB2 and** ***A. vaginae*** **(n = 339).**
(DOC)

Table S6 **Summary of major findings relating BV candidate organisms to BV status and sexual risk behaviours.**
(DOC)
