# Supplementary material for: Bacterial Vaginosis (BV) Candidate Bacteria: Associations with BV and Behavioural Practices in Sexually-Experienced and Inexperienced Women
Source: PLoS One. 2012 Feb 17;7(2):e30633. doi: 10.1371/journal.pone.0030633 (PMC3281856; doi:10.1371/journal.pone.0030633)
Supplement: Table S1 — Demographic and behavioural characteristics of study population. (DOC) [file pone.0030633.s001.doc]

**Table S**1. Demographic and behavioural characteristics of study population

| **Characteristic** | **No. (%) of participants in FUSS n=193** | **No. (%) of participants in clinic study n=146** | **P value** | **No. (%) of participants total n=339** |
| --- | --- | --- | --- | --- |
| **NF** | 169 (88) | 64 (44) | <0.001 | 233 (69) |
| **BV** | 24 (12) | 82 (56) |  | 106 (31) |
| ***Demographics*** |  |  |  |  |
| ***Age*** |  |  |  |  |
| **<21 years** | 154 (80) | 5 (3) | <0.001 | 159 (47) |
| **≥21 years** | 39 (20) | 141 (97) |  | 180 (53) |
| **Country of birth** |  |  |  |  |
| **Australia** | 140 (73) | 106 (73) | 0.8 | 246 (73) |
| **Other** | 53 (28) | 38 (24) |  | 91 (27) |
| ***Lifetime sexual history variables*** | | | | |
| **Ever had vaginal sex** |  |  |  |  |
| **No** | 82 (43) | 0 | <0.001 | 82 (24) |
| **Yes** | 111 (58) | 146 (100) |  | 257 (76) |
| **Lifetime vaginal sex partners** | | | | |
| **0** | 82 (43) | 0 | <0.001 | 82 (24) |
| **1-10** | 98 (51) | 17 (12) |  | 115 (34) |
| **>10** | 13 (7) | 128 (88)^a^ |  | 141 (42) |
| **Ever had anal sex^b^** | | | | |
| **No** | 155 (80) | 95 (66) | 0.002 | 250 (74) |
| **Yes** | 39 (20) | 50 (35)^a^ |  | 88 (26) |
| **Ever had receptive oral or digital-vulvovaginal sex** | | | | |
| **No** | 58 (30) | 0 | <0.001 | 58 (17) |
| **Yes** | 135 (70) | 146 (100) |  | 281 (83) |
| **Ever used a sex toy with a sex partner** | | | | |
| **No** | 155 (80) | 131 (90) | 0.02 | 286 (84) |
| **Yes** | 38 (20) | 15 (10) |  | 53 (16) |
| ***Recent sexual history*** | | | | |
| **Oral sexual contact with a women last 12 months** | | | | |
| **No** | 187 (97) | 113 (77) | <0.001 | 300 (89) |
| **Yes** | 6 (3) | 33 (23) |  | 39 (12) |
| **Oral sexual contact with a man last 12 months** | | | | |
| **No** | 95 (50) | 7 (5) | <0.001 | 103 (30) |
| **Yes** | 97 (50) | 138 (95)^a^ |  | 235 (70) |
| **Oral sex > once weekly** | | | | |
| **No** | 178 (92) | 48 (33) | <0.001 | 226 (67) |
| **Yes** | 15 (8) | 98 (67) |  | 113 (33) |
| **UPVSI in the last 12 months** |  |  |  |  |
| **No** | 102 (53) | 3 (2) | <0.001 | 105 (31) |
| **Yes** | 91 (47) | 143 (98) |  | 234 (69) |
| **Vaginal sex > once weekly** | | | | |
| **No** | 146 (76) | 38 (26) | <0.001 | 184 (54) |
| **Yes** | 47 (24) | 108 (74) |  | 155 (46) |
| **Current/most recent partner circumcised^c^** | | | | |
| **No** | 78 (40) | 35 (24) | 0.001 | 113 (33) |
| **Yes** | 27 (14) | 38 (26) |  | 65 (19) |
| ***Other behavioural variables*** | | | | |
| **Current oral contraceptive pill use** | | | | |
| **No** | 120 (62) | 80 (55) | 0.2 | 200 (59) |
| **Yes** | 73 (38) | 66 (45) |  | 139 (41) |
| **Ever douched vaginally** | | | | |
| **No** | 156 (81) | 121 (83) | 0.6 | 277 (82) |
| **Yes** | 37 (19) | 25 (17) |  | 62 (18) |
| **Current smoker** | | | | |
| **No** | 178 (92) | 72 (50) | <0.001 | 250 (74) |
| **Yes** | 15 (8) | 73 (50)a |  | 88 (26) |
| **Abnormal vaginal discharge and/or odour^d^** | | | | |
| **Nil to Mild** | 48 (25) | 10 (7) | <0.001 | 58 (17) |
| **Moderate** | 108 (56) | 73 (52) |  | 181 (54) |
| **Severe** | 37 (19) | 57 (41) |  | 94 (28) |

Footnotes: No.=number, NF=normal flora, BV=bacterial vaginosis, ^a^ missing data=2, ^b^FUSS =anal sex "ever", clinic =anal sex "last 12months", Oral sex = receptive oral sex, UPVSI=unprotected vaginal sex, ^c^161(47%) patients did not provide this information comprising 88 (46%) from the FUSS cohort and 73 (50%) from the clinic study, **^d^** data missing = 6 from Clinic population.
