# Supplementary material for: Bacterial Vaginosis (BV) Candidate Bacteria: Associations with BV and Behavioural Practices in Sexually-Experienced and Inexperienced Women
Source: PLoS One. 2012 Feb 17;7(2):e30633. doi: 10.1371/journal.pone.0030633 (PMC3281856; doi:10.1371/journal.pone.0030633)
Supplement: Table S2 — Bacterial vaginosis candidate organisms in women with normal flora and BV. (DOC) [file pone.0030633.s002.doc]

**Table S**2. Bacterial vaginosis candidate organisms in women with normal flora and BV

| **Candidate Organism** | **NF n=233** | **BV n=106** | **Crude ORs (95%CI)** | **Adjusted ORs (95%CI)^a^** | **Adjusted p value** |
| --- | --- | --- | --- | --- | --- |
| ***Megasphaera* type I (%)** | 9 (4) | 81 (76) | 83 (36‑167) | 15.4 (5.9-40.2) | <0.001 |
| ***G. vaginalis* (%)** | 114 (53) | 103 (97) | 36 (11‑111) | 4.1 (1.0-17.2) | 0.05 |
| ***Sneathia* spp*.* (%)** | 17 (7) | 78 (74) | 36 (19‑67) | 2.5 (0.5-13.1) | 0.3 |
| ***Leptotrichia* spp*.* (%)** | 18 (8) | 77 (73) | 31 (59‑17) | 1.9 (0.4-9.8) | 0.5 |
| **BVAB1 (%)** | 0 | 7 (7) | - | - | - |
| **BVAB2 (%)** | 12 (14) | 73 (69) | 40 (20‑83) | 4.2 (1.4-12.0) | 0.008 |
| **BVAB3 (%)** | 2 (1) | 17 (16) | 22 (5‑100) | 1.4 (0.1-13.8) | 0.8 |
| ***A. vaginae*^b^ (%)** | 143 (62) | 103 (99) | 63 (9‑500) | 15.3 (1.7-138.4) | 0.02 |
| ***L. crispatus* (%)** | 164 (78) | 47 (44) | 0.3 (0.2‑0.5) | - | - |

**^a^** adjusted for all organisms except BVAB1 and *L. crispatus*, **^b^** data missing=3, NF= normal flora (Nugent score 0-3), BV = bacterial vaginosis (Nugent score 7-10), N=number.
