# Supplementary material for: Bacterial Vaginosis (BV) Candidate Bacteria: Associations with BV and Behavioural Practices in Sexually-Experienced and Inexperienced Women
Source: PLoS One. 2012 Feb 17;7(2):e30633. doi: 10.1371/journal.pone.0030633 (PMC3281856; doi:10.1371/journal.pone.0030633)
Supplement: Table S3 — Associations between bacterial vaginosis candidate organisms and level of sexual exposure in women with normal flora and BV. (DOC) [file pone.0030633.s003.doc]

**Table S3. Associations between bacterial vaginosis candidate organisms and level of sexual exposure in women with normal flora and BV**

|  |  | | ***Megasphaera* type I (%)** | ***Sneathia* (%)** | ***Leptotrichia* (%)** | ***G. vaginalis* (%)** | **BVAB1 (%)** | **BVAB2 (%)** | **BVAB3 (%)** | ***A. vaginae* (%)** |
| --- | --- | --- | --- | --- | --- | --- | --- | --- | --- | --- |
| **Women with NV (n=233)** | **No sexual contact ever (n=58)** | | 1 (2) | 1 (2) | 1 (2) | 16 (28) | 0 | 0 | 0 | 41 (71) |
|  | **Protected sex or non-coital (n=40)^a^** | | 2 (5) | 2 (5) | 3 (8) | 18 (45) | 0 | 0 | 0 | 25 (63) |
|  | **Unprotected vaginal sex ever (n=135)** | | 6 (4) | 14 (10) | 14 (10) | 80 (59) | 0 | 12 (9) | 2 (2) | 77 (58) |
|  |  | **p for trend** | 0.6 | 0.04 | 0.06 | <0.001 |  | 0.009 |  | 0.2 |
|  | **LVSP^c^** | |  |  |  |  |  |  |  |  |
|  |  | **0** | 2/79 (3) | 3/79 (4) | 3/79 (4) | 24/79 (30) | 0/79 | 0/79 | 0/79 | 53/79 (67) |
|  |  | **1-10** | 1/82 (1) | 4/82 (5) | 3/82 (4) | 41/82 (50) | 0/82 | 6/82 (7) | 0/82 | 56/82 (68) |
|  |  | **>10** | 6/72 (8) | 10/72 (14) | 12/72 (17) | 49/72 (68) | 0/72 | 6/72 (8) | 2/73 (3) | 34/72 (48) |
|  |  | **p for trend** | 0.1 | 0.03 | 0.006 | <0.001 |  | 0.2 |  | 0.02 |
| **Women with BV (n=106)** | **No sexual contact ever (n=0)*** | | - | - | - | - | - | - | - | - |
|  | **Protected sex or non-coital (n=7)^b^** | | 4 (57) | 1 (14) | 1 (14) | 7 (100) | 0 | 1 (14) | 0 | 1 (14) |
|  | **Unprotected vaginal sex ever (n=99)** | | 77 (78) | 77 (78) | 76 (77) | 96 (97) | 7 (7) | 72 (73) | 17 (17) | 97 (100) |
|  |  | **p for trend** | 0.4 | 0.001 | 0.002 | 1.0 | 1.0 | 0.003 | 0.6 | 0.07 |
|  | **LVSP^c^** | |  |  |  |  |  |  |  |  |
|  |  | **0** | 2/3 (66) | 1/3 (33) | 1/3 (33) | 3/3 (100) | 0/3 | 0/3 | 0/3 | 2/3 (67) |
|  |  | **1-10** | 21/33 (64) | 17/33 (52) | 16/33 (49) | 31/33 (94) | 1/33 (3) | 18/33 (55) | 4/33 (12) | 33/33 (100) |
|  |  | **>10** | 57/69 (83) | 60/69 (87) | 59/69 (86) | 69/69 (100) | 6/69 (9) | 54/69 (78) | 13/69 (19) | 67/67 (100) |
|  |  | **p for trend** | 0.07 | <0.001 | <0.001 | 0.3 | 0.3 | 0.004 | 0.3 | 0.7 |

* No cases of BV in women with no history of sexual contact ever, ^a^ 24 women reported only ever engaging in non-coital sexual practices with another individual and 16 women reported 100% condom use for vaginal sex in their lifetime, **^b^** 4 women reported 100% condom use always and 3 reported only ever engaging in non-coital sex, ^c^ LVSP = Lifetime Vaginal Sexual Partners
