# Supplementary material for: Bacterial Vaginosis (BV) Candidate Bacteria: Associations with BV and Behavioural Practices in Sexually-Experienced and Inexperienced Women
Source: PLoS One. 2012 Feb 17;7(2):e30633. doi: 10.1371/journal.pone.0030633 (PMC3281856; doi:10.1371/journal.pone.0030633)
Supplement: Table S4 — Odds ratios for BV-COs behavioural risks factors adjusted for BV status (n = 339). (DOC) [file pone.0030633.s004.doc]

**Table S4. Odds ratios for BV-COs behavioural risks factors adjusted for BV status ^a^ (n=339)**

| **BV risk variable** | ***Megasphaera* type I** | ***G. vaginalis*** | ***Sneathia* spp*.*** | ***Leptotrichia* spp*.*** | **BVAB1** | **BVAB2** | **BVAB3** | ***A. vaginae*** |
| --- | --- | --- | --- | --- | --- | --- | --- | --- |
| **LSP^b^ >10** | 3.2 (1.5-6.9) | 3.4 (1.9-6.0) | 5.0 (2.5-10.0) | 5.9 (3.0-11.8) | 3.3 (0.4-28.8) | 3.1 (1.5-6.2) | 2.7 (0.8-8.8) | 0.5 (0.3-0.8) |
|  | p=0.003 | p<0.001 | p<0.001 | p<0.001 | p=0.3 | p=0.001 | p=0.01 | p=0.01 |
| **Vaginal sex >weekly** | 0.7 (0.3-1.7) | 2.1 (1.3-3.7) | 2.5 (1.3-4.8) | 1.8 (0.9-3.5) | 2.7 (0.3-23.7) | 1.7 (0.8-3.3) | 5.5 (1.9-25.1) | 0.8 (0.4-1.3) |
|  | p=0.5 | p=0.005 | p=0.008 | p=0.08 | p=0.4 | p=0.2 | p=0.03 | p=0.3 |
| **Oral sex >weekly** | 0.9 (0.4-2.0) | 3.0 (1.6-5.5) | 2.1 (1.1-4.1) | 2.1 (1.1-4.1) | 2.2 (0.4-11.7) | 1.0 (0.5-2.1) | 2.4 (0.8-6.8) | 0.6 (0.3-1.1) |
|  | p=0.9 | p=0.001 | p=0.03 | p=0.02 | p=0.4 | p=0.9 | p=0.1 | p=0.07 |
| **Smoker** | 2.0 (0.9-4.4) | 2.0 (1.0-3.9) | 1.8 (0.9-3.6) | 1.8 (0.9-3.6) | 0.4 (0.1-2.3) | 1.4 (0.7-2.9) | 0.9 (0.3-2.6) | 0.8 (0.4-1.6) |
|  | p=0.09 | p=0.05 | p=0.1 | p=0.1 | p=0.3 | p=0.4 | p=0.9 | p=0.5 |
| **Age >20 years** | 1.3 (0.6-3.0) | 1.5 (0.9-2.6) | 2.3 (1.1-4.6) | 2.7 (1.3-5.3) | undefined | 2.9 (1.4-6.1) | 8.2 (1.0-64.4) | 0.6 (0.4-1.1) |
|  | p=0.5 | p=0.1 | p=0.02 | p=0.006 |  | p=0.005 | p=0.05 | p=0.1 |
| **WSW** | 5.8 (1.8-18.7) | 2.7 (0.9-7.7) | 1.9 (0.8-4.9) | 3.1 (1.2-7.9) | 1.5 (0.3-8.2) | 1.1 (0.4-2.7) | 1.5 (0.5-4.8) | 1.1 (0.4-3.1) |
|  | p=0.003 | p=0.07 | p=0.2 | p=0.02 | p=0.7 | p=0.9 | p=0.5 | p=0.9 |
| **UPVSI last 12 months** | 1.9 (0.6-5.7) | 2.6 (1.6-4.5) | 6.9 (2.2-21.8) | 5.2 (1.8-14.7) | undefined | 26.5 (3.4-206.5) | undefined | 0.7 (0.4-1.2) |
|  | p=0.2 | p<0.001 | p=0.001 | p=0.002 |  | p=0.002 |  | p=0.2 |

Footnotes: **^a^** Presence or absence of BV was included in the regression model. WSW=women who received oral sex from a woman in the last 12 months, oral sex = receptive oral sex, UPVSI=unprotected vaginal sex. Only variables that were not highly correlated were included in the model. ^b^ LSP=Lifetime Sexual Partners
