# Supplementary material for: Bacterial Vaginosis (BV) Candidate Bacteria: Associations with BV and Behavioural Practices in Sexually-Experienced and Inexperienced Women
Source: PLoS One. 2012 Feb 17;7(2):e30633. doi: 10.1371/journal.pone.0030633 (PMC3281856; doi:10.1371/journal.pone.0030633)
Supplement: Table S5 — Logistic regression analysis for BV and BV risk factors and Megasphaera type I, G. vaginalis , BVAB2 and A. vaginae (n = 339). (DOC) [file pone.0030633.s005.doc]

**Table S5. Logistic regression analysis for BV and BV risk factors and *Megasphaera* type I*, G. vaginalis*, BVAB2 and *A. vaginae*** (n=339)

| **BV risk variable** | ***Megasphaera* type I AOR (95%CI)** | ***G. vaginalis* AOR (95%CI)** | **BVAB2 AOR (95%CI)** | ***A. vaginae* AOR (95%CI)** |
| --- | --- | --- | --- | --- |
| **LSP >10** | 2.9 (1.0-8.3) | 2.3 (0.9-5.8) | 1.8 (0.7-4.9) | 0.4 (0.2-1.1) |
|  | p=0.04 | p=0.07 | p=0.2 | p=0.07 |
| **Vaginal sex > weekly** | 0.7 (0.2-2.2) | 0.8 (0.3-1.7) | 1.3 (0.5-3.6) | 1.2 (0.5-2.5) |
|  | p=0.5 | p=0.5 | p=0.6 | p=0.7 |
| **Oral sex >weekly** | 0.6 (0.2-1.8) | 2.1 (0.8-5.6) | 0.3 (0.1-0.9) | 0.7 (0.3-2.0) |
|  | p=0.4 | p=0.1 | p=0.03 | p=0.5 |
| **Smoker** | 1.6 (0.7-3.8) | 1.1 (0.5-2.4) | 1.0 (0.4-2.6) | 1.2 (0.5-2.6) |
|  | p=0.3 | p=0.9 | p=1.0 | p=0.7 |
| **Age >20 years** | 0.6 (0.2-1.9) | 0.6 (0.3-1.3) | 2.1 (0.7-6.1) | 1.0 (0.5-2.5) |
|  | p=0.4 | p=0.2 | p=0.1 | p=1.0 |
| **WSW** | 4.4 (1.2-16.4) | 1.2 (0.4-4.1) | 0.8 (0.3-2.2) | 2.0 (0.6-6.3) |
|  | p=0.03 | p=0.7 | p=0.6 | p=0.2 |
| **UPVSI last 12 months** | 1.8 (0.5-7.0) | 1.9 (0.9-3.9) | 22.5 (2.6-196.0) | 1.0 (0.5-2.2) |
|  | p=0.4 | p=0.09 | p=0.005 | p=1.0 |
| **BV status** | 81.4 (31.7-209.4) | 37.4 (8.7-160.6) | 30.8 (13.6-69.8) | 80.9 (10.8-605.2) |
|  | p<0.001 | p<0.001 | p<0.001 | p<0.001 |

Footnotes: Behavioural variables included into logistic regression analysis are those listed in addition to BV status and were not highly correlated. WSW=women who received oral sex from a woman in the last 12 months, oral sex = receptive oral sex, LSP = Lifetime Sexual Partner, UPVSI=unprotected vaginal sex
