# Supplementary material for: Bacterial Vaginosis (BV) Candidate Bacteria: Associations with BV and Behavioural Practices in Sexually-Experienced and Inexperienced Women
Source: PLoS One. 2012 Feb 17;7(2):e30633. doi: 10.1371/journal.pone.0030633 (PMC3281856; doi:10.1371/journal.pone.0030633)
Supplement: Table S6 — Summary of major findings relating BV candidate organisms to BV status and sexual risk behaviours. (DOC) [file pone.0030633.s006.doc]

**Table S6. Summary of major findings relating BV candidate organisms to BV status and sexual risk behaviours**

| **Characteristic** | | **Bacteria with Characteristic** | **Bacteria without Characteristic** |
| --- | --- | --- | --- |
| **BV-CO absent/rare in women with no sexual experience ^a^** | | BVAB1 | *A. vaginalis* |
|  |  | BVAB2 | *G. vaginalis* |
|  |  | BVAB3 |  |
|  |  | *Leptotrichia* spp. |  |
|  |  | *Megasphaera* type I |  |
|  |  | *Sneathia* spp. |  |
| **BV-CO that demonstrate an association with: ^b^** | | BVAB1 | *A. vaginalis* |
|  | **i) increased level of sexual activity** | BVAB2 | BVAB1 |
|  | **and/or** | BVAB3 |  |
|  | **ii) increased numbers of LSP** | *G. vaginalis* |  |
|  |  | *Leptotrichia* spp. |  |
|  |  | *Megasphaera* type I |  |
|  |  | *Sneathia* spp. |  |
| **BV-COs with an independent association with BV disease by multivariable analysis ^c^** | | *A. vaginalis* | BVAB1 |
|  |  | BVAB2 | BVAB3 |
|  |  | *G. vaginalis* | *Leptotrichia* spp. |
|  |  | *Megasphaera* type I | *Sneathia* spp. |
| **BV-COs that are: ^d^** | | BVAB2 | *A. vaginalis* |
|  | **i) absent/rare in sexually-inactive women** | *Megasphaera* type I | BVAB1 |
|  | **ii) demonstrate sexual risk behavioural characteristics** |  | BVAB3 |
|  | **iii) independently associated with BV disease** |  | *G. vaginalis* |
|  |  |  | *Leptotrichia* spp. |
|  |  |  | *Sneathia* spp. |

BV-CO=bacterial vaginosis candidate organism, UPVSI=unprotected vaginal sex, LSP=lifetime numbers of vaginal sex partners, STI=sexually transmitted infection, ^a^ BVAB1, 2 & 3 absent in women without a history of UPVSI. *Leptotrichia* spp.. *Megasphaera* type I and Sneathia spp. rarely detected in women without a history of sexual contact (1/58), ^b^ BVAB1 uncommon and no associations could be determined with meaningful statistical power. *Megasphaera* type I significantly associated with >10 LSP when adjusted for BV status, but not in analysis stratified for BV status, ^c^ BVAB1 uncommon and no association could be determined with meaningful statistical power, d If BV is an STI, one would expect any causative organisms to fulfil these 3 criteria.
